# Near Field Communication-based Agricultural Management Service Systems for Family Farms

**DOI:** 10.3390/s19204406

**Published:** 2019-10-11

**Authors:** Xue-fen Wan, Tao Zheng, Jian Cui, Fan Zhang, Zi-qian Ma, Yi Yang

**Affiliations:** 1 Hebei IoT Monitoring Engineering Technology Research Center/Computer College, North China Institute of Science and Technology, Langfang 065201, China; calmerd@ncist.edu.cn; 2 School of Economics and Management, Yanshan University, Qinhuangdao 066004, China; 3 School of Cyber Science and Technology, Beijing University of Aeronautics and Astronautics, Beijing 100083, China; cuijianw@buaa.edu.cn; 4 College of Information Science and Technology, Donghua University, Shanghai 201620, China; 2181287@mail.dhu.edu.cn (F.Z.); mzq0920@163.com (Z.-q.M.)

**Keywords:** NFC, smart agriculture, family farm, smartphone, Android app, programmable system-on-chip

## Abstract

This paper presents an agricultural management service system that aims to meet the needs of Internet of Things (IoT) information upgrades in China’s family farms. The proposed agricultural management service system consists of Near Field Communication (NFC) tags, in-field service nodes, and smartphones. NFC tags are used as the core identifier of various agricultural management elements. The in-field service node, which is based on a programmable system-on-chip with intellectual property cores (IP core), supports distributed agriculture device management and smartphone operations. Smartphones in the proposed system include the management assistant application (app) and management service app, which are designed for agricultural management support functions and agricultural management application requirements. Through this system, the needs of diverse agricultural management practices can be effectively satisfied by a unified system structure. The practical results show that the design can be used to construct diversified agricultural IoT information application service systems simply and effectively, and it is especially suitable for Chinese family farm operators who are implementing IoT information upgrades for smart agriculture.

## 1. Introduction

In today’s world, especially in developing countries, there is an increasing shortage of agricultural resources [1,2,3]. In China, this problem is particularly serious [4,5]. Statistics have revealed that the average cultivated land area of Chinese farmers is below 0.2 hectares, and the per capita water resources are about 2000 cubic meters and continue to decrease every year. In addition, the loss of rural labor due to off-farm work, among other reasons, is a critical issue. Therefore, effective means of improving the utilization of agricultural resources are urgently needed [6,7]. Family farms are a form of rural production organization and are transitioning from traditional agriculture to modern agriculture; in other words, non-standardized traditional family farming methods are being changed to standardized enterprise farming methods [7,8]. The development of family farms helps integrate production resources and increase agricultural productivity. On the one hand, their processes are similar to the traditional family-based agricultural production methods. On the other hand, family farms have made breakthroughs in production-scale and specific planting methods, and they are in the embryonic form of agricultural enterprise and large-scale production. In addition to ensuring the income and employment of the rural population and realizing the efficient use of agricultural resources, the family farm method facilitates the modernization of agricultural production by implementing agricultural science and technology. Therefore, family farms are increasingly becoming one of the most popular forms of agricultural production and management in developing countries such as China [9,10,11]. However, establishing processes that optimize the efficiency of resource utilization in family farms is a problem that requires urgent solutions. The introduction of agricultural IoT management systems is an effective means of improving the utilization of agricultural resources [12,13,14,15]. If the backbone of the family farm management process is built on such technology, then achieving the all-around optimization of agricultural resources is expected [16,17].

An important part of establishing a family farm IoT management service system is the selection of a suitable application information platform [18,19,20]. The popularity of equipment in rural China is the key criterion for platform selection. Only highly popular systems reflect the daily habits and processes of the majority of agricultural operators, and such systems can be promoted rapidly and at low cost in rural China. With the support of appropriate infrastructure construction, the popularization of smartphones will further promote the development of rural information management technology [21]. In recent years, smartphones have been quickly and widely adopted in China. In 2018, there were more than 1.5 billion mobile phone user accounts in China, and the average number of mobile phones per 100 people in China reached 112.3. Most of them were smartphones with an Android operating system. In rural China, smartphones have replaced computers, televisions, and other information equipment and have become the most prevalent information devices in rural areas. In addition, research has shown that people’s mobility between cities and rural areas (most frequently for off-farm work and labor export) has further accelerated smartphone-based e-commerce, online finance, social networking, and information services in rural China [22]. This has further enhanced the popularity of smartphones in rural China. Therefore, choosing the smartphone as the core platform of an information management system for family farms is highly consistent with the current situation in rural China. Smartphones have strong computing and storage capabilities, enhanced communication capabilities, an easy-to-use human–machine interface, excellent image acquisition capabilities, and a relatively low cost. They have gained a certain degree of use in agricultural fields for purposes such as agricultural information management systems [23,24,25,26]. The functional support of smartphones is another aspect that makes them very suitable cores of the information management system platform for family farms in rural China.

In the design of a smartphone-based information management service system for Chinese family farms, it is necessary to focus on agricultural process management, farm product business management, and value-added services. In the management of agricultural processes on family farms, it is necessary to focus on the management needs of agricultural electromechanical equipment, agricultural IoT equipment, and agricultural employees. Implementation of different precise management solutions for specific agricultural processes is expected to support the realization of accurate macro- and micro management of agricultural processes and improve the utilization of agricultural resources [15,18,19]. In addition, the quick connection between smartphones and IoT terminals needs to be considered. The management of farm products requires the traceability of the attributes, production processes, and sales processes of farm products. In particular, with the emphasis on food safety in Chinese society in recent years, the ability to backtrack pesticides and fertilizers in the production process should be emphasized during the design of agricultural information systems. Effective labeling of farm product attributes can help improve quality and promote the sale of farm products [15,17]. For agricultural value-added services, the immersive experience of the agricultural process is considered to have a bright future. In particular, agricultural tourism and agricultural education for children and adolescents are expected to benefit family farm operators while also bringing value to society [27]. In the design of IoT management systems for family farms, it is also necessary to add functions that are tailored to their specific needs. In addition, a variety of novel IoT devices are emerging, which also opens up new possibilities for agricultural management service systems [28,29,30]. Notably, because information management differs among Chinese family farms, the information management systems will also show great variability among different farms. This is a significant challenge for designers.

In the distributed agricultural management service system for Chinese family farms, the core is to have a suitable information storage unit as the core of agricultural processing management and service. Radio Frequency Identifications (RFIDs) have been promoted and applied in the management service systems for years [31,32,33,34,35]. In the agricultural field, RFIDs have also been widely used in agricultural product supply chain management, aquaculture management, food safety management and other fields [36,37,38,39,40,41], but is difficult to integrate RFIDs with smartphones. In the family farm management in China, if RFID technology is used, additional infrastructure, such as RFID readers, will need to be added to agricultural work sites, agricultural logistics, and agricultural value-added services. Compared with the technology which is directly based on smartphone, the cost and ease of use of RDID have a certain degree of defects. Especially in some areas for smart agricultural tourism, it is difficult for tourists to use RFID equipment without training, which will also bring inconvenience to tourists and affect their tourist experience. Barcodes and Quick Response Code (QR code) have been widely used in smartphone-related fields [42,43,44]. However, it is difficult to avoid the staining of barcodes or QR codes during the process of agricultural operation (especially for soil, foliage, etc.), and it is difficult to modify the information in barcodes or QR codes in the agricultural process, so it is difficult to apply barcodes or QR codes in the whole agricultural process of the family farm.

Near field communication technology (NFC) has been frequently used for diverse applications, such as intelligent transportation, industrial automation, smart homes, and other fields. The popularity of NFC is due to advantages such as its low cost, convenience, reliability, and ability to integrate with smartphones [45,46,47,48,49]. Based on NFC, it is possible to build management services with different sizes system for many fields [50,51,52,53]. Among these management service systems, NFC tags serve as the core information storage unit [54,55,56,57,58,59]. However, in such management information system based on NFC tags and smartphones, the apps have a high degree of relevance to the application scenario. Because the demand for Chinese family farms varies widely, if the app is developed in the wrong way, the development cycle and cost are unacceptable. In addition, if an agricultural management service system based on NFC tags and smartphones is to be established, it is necessary to have an appropriate hardware design to construct an effective information channel for specific agricultural equipment management. However, there are many kinds of equipment in agricultural processes in Chinese family farms, so the hardware design faces great challenges too.

On the basis of needs of Chinese family farms, an NFC-based agricultural management system is presented. Applying NFC-based design to the information management system for family farms will facilitate the realization of the smartphone-based distributed information platform, and it is also expected to support various types of agricultural operations at a lower cost. The use of NFC can also improve the management of the entire process of agricultural production and sales, and it is also easy to provide diversified agricultural value-added services. Compared with the traditional computer-based agricultural management system [60,61], this system uses smartphones and NFC as the core management information platform, so it has lower cost and strong distributed management capability. Moreover, with smartphones, which are widely used in rural China, farmers with lower education can manage agriculture processes directly in the agricultural field through e -to-understand interfaces of smartphones. They are able to combine their own agricultural management experience with the in-field scenarios and the distributed management effect is stronger and directly and with the NFC and smartphone platform, the entire agricultural process can be included in the unified management service system. Especially in the fields of agricultural value-added services (such as agricultural tourism services), which are paid less attention in agricultural management systems in the past, this system has strong practicability. Secondly, the In-field service node adopts a programmable system-on-chip to obtain a unified hardware structure, so that it can adapt to the diverse connection and management requirements in Chinese family farms. By simply adjusting the IP core, the diverse needs can be satisfied on a unified hardware platform without hardware changing. Through this design, information islands can be effectively eliminated, and various agricultural devices can be incorporated into the IoT management service system with low cost quickly and conveniently. Compared with other smartphone-based agricultural management systems, the system separates application requirements from management support requirements, respectively Android management assistance application (MA App) and Android management service application (MS App). Through the MA App, it is possible to effectively manage the differentiation of the underlying hardware structure and the difference in information service requirements existing in different family farms, and to provide a service with greater differentiation on the unified support structure. This is also a significant advantage of the system.

## 2. System Overview

**Figure 1:**
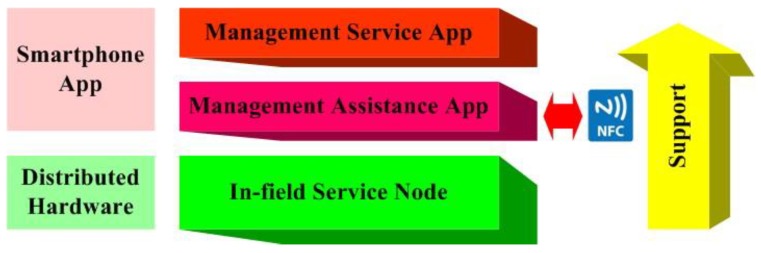
Function schematic of presented system.

The system presented in this paper is designed to meet the needs of agricultural process management, farm product management, and value-added services on Chinese family farms. The system has a distributed structure. Smartphones and the terminal nodes of distributed agricultural information are used to implement the family farm’s daily information management operations, which are based on the diverse information management functions of NFC tags. The deployment of NFC tags can lead to the realization of agricultural-specific and refined information services within the scope of family farm. Such processes include agricultural equipment management, agricultural planting variety management, agricultural planting process records, agricultural product market management, agricultural human resource management, and agricultural value-added service management. The function schematic of presented system is shown in Figure 1.

The proposed system consists of NFC tags, an in-field service node, an Android MA App and Android MS App. NFC tags are placed on the surface of agricultural devices, as well as in in-field service access positions, agricultural process-tracking positions, and agricultural product packages. With the deployment of these NFC tags, the corresponding agricultural information management service can be implemented. The information stored in the NFC tag is encoded data. The coding method is uniformly set according to the specific needs of the information structure of the family farm.

**Figure 2:**
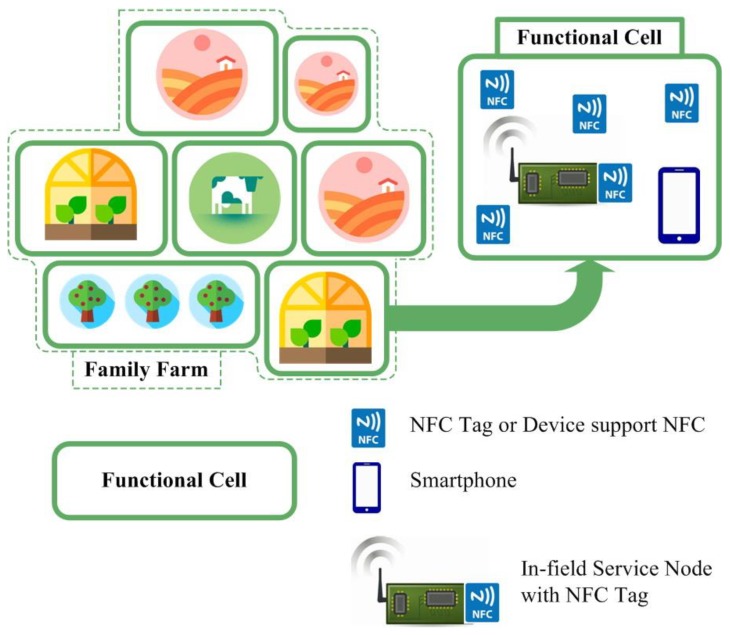
Schematic of the function cells in family farm.

The in-field service node is a distributed agricultural information terminal node and based on a programmable system-on-chip (PSoC). The node is placed in a location where family farms need information management. The MA App is responsible for encapsulating the hardware function of in-field service nodes and Android operations for common agricultural information management, and it also supports the MS App. The MA App is a bridge between the in-field service node hardware functional structure and differentiated management application requirements of the family farm. The MS App’s functions are built on the MA App, and it is a bridge between smartphone users and the common agricultural information management operations. It should be emphasized that only the MA App performs NFC reading and writing and other NFC-based interaction information operations in this system. The NFC-based information interaction between the in-field service node and the MS App needs to be performed by the MA App. The schematic of the function cells in family farm is shown in Figure 2.

Using this structure, we divided the NFC tag, system hardware, information management support functions, and application requirements into separate parts. With the unified NFC tag, the in-field service node hardware structure, and the MA App, developers are able to meet the diverse application needs of different family farm agricultural scenarios. The structure of the system can also meet the differentiated business needs of Chinese family farms and accounts for the complexity of system development and development efficiency. This approach allows system developers to provide a diverse information management system quickly and cost-effectively by using a unified platform for family farms.

In order to provide in-field information management services efficiently, the family farm was divided into cells with different functions according to positions and agricultural functions. Each function cell has a unique identification (ID) and is treated as an independent information unit. This division has a high degree of flexibility that can meet the needs of distributed agricultural information management and support centralized agricultural information management. In addition, it is especially suitable for the mid- and long-term gradual and partial informationization involved in the information upgrade of some of today’s Chinese family farms. An in-field service node is installed in each function cell, and the ID of this in-field service node is the same as the ID of the function cell. This in-field service node serves as the information management access point for the function cell and supports the information management service with the smartphone. Both the in-field service node and the smartphone follow the NFC-centric information operation flow. Each function cell is in the Bluetooth coverage range of its corresponding in-field service node, so a smartphone located inside the function cell can easily access the in-field service node. There are also multiple NFC tags in the function cell for implementing various agricultural information management functions. Agricultural electromechanical equipment and agricultural IoT equipment can be added to the in-field service node through smartphone-based NFC tag operations. The smartphone also uses the in-field service node as a bridge to implement the daily information management of the function cell with the aid of the NFC tag. In each function cell, the agricultural processes are also recorded through NFC, the in-field service node, and the smartphone. Related elements are added to or revised in the NFC tags within the agricultural processes. Agricultural value-added services are also completed by NFC-based operations with the smartphone and in-field service node. The agriculture information of each function cell can also be sent to the host computer through the LoRa low-power wide-area network (LoRa LPWAN) when centralized agricultural assistance management is needed.

**Figure 3:**
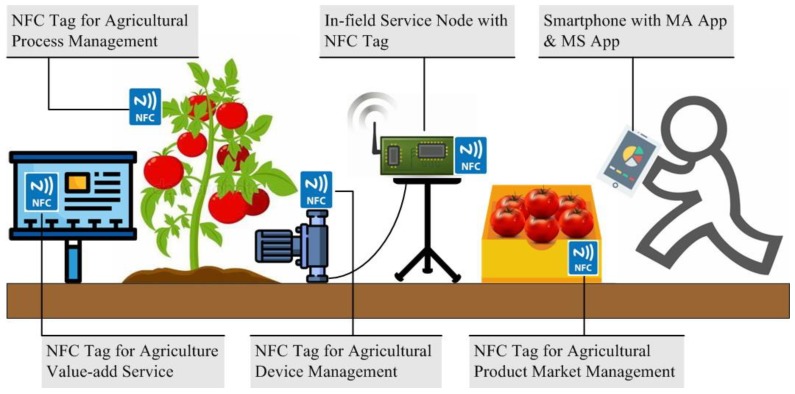
NFC tags and related functions in tomato greenhouse.

As an example, suppose that a function cell consists of only one small tomato greenhouse. This greenhouse needs to meet the needs of both intelligent tomato planting and agricultural tourism. The in-field information service is validated by installing an in-field service node in the greenhouse. After the installation is completed, the smartphone can access the in-field service node through Bluetooth by scanning the NFC tag of the in-field service node. Thereafter, the smartphone MA App obtains the information of the in-field service node and is responsible for functional operations related to the in-field service node. If an agricultural electromechanical device or an agricultural IoT device needs to be connected to the in-field service node, the farmer can use the MS App to obtain the device parameters in the NFC tag through the MA App. Then, the device parameters in the NFC tag are passed to the in-field service node, and the in-field service node automatically implements the data connection according to the device parameters in the NFC tag through the IP function module preset within the PSoC. In daily agricultural process management, tomato varieties, dates of fruiting, dates of maturity, and daily management information (such as fertilizer application time and type) are also written by the user to the in-field service node (macro agricultural process parameters) and the NFC tag near plant (micro agricultural process parameters) via the smartphone. For human resource management on the family farm, the information exchange between the farm’s smartphone and the in-field service node can be included and later referenced. When the tomatoes are ripe and picked, the farmers use the smartphone to access the in-field service node and NFC tag near the tomato plants to retrieve information about the entire agricultural process. This information is then written to the NFC product label on the tomato package. This process information, together with the picking date, shelf life, selling price, and logistics information, among other data, constitutes the business information in the NFC tag of the tomato. The picking process in agricultural tourism is implemented by setting NFC tags at multiple locations in the greenhouse to assist picking. These NFC tags contain the characteristics of the agricultural products and the sales information. Tourists can also participate in the daily greenhouse management process through the tourism app (the MS App with reduced function) combined with NFC tag operations. If there is a need for centralized management at the same time, the in-field service node sends the changed information to the host computer through the LoRa data channel after each smartphone access session (farmer or visitor) is completed. The NFC tags and related functions in tomato greenhouse is shown in Figure 3.

## 3. NFC Tag Design

As discussed above, NFC tags play a vital role in the distributed agricultural management systems that are applied to family farms. The content of the NFC label used in this system needs to be unified, and it is oriented to the whole process of production management of family farms. The information such as farming process and agricultural value-added services is difficult to use EPCglobal related standards because it involves more independent production processes and related objects of farmers’ description. The EPCglobal-related standards also lack Mandarin/Chinese support for intuitive reading and writing of NFC tags. In addition, due to the requirements of family farms and service providers for independent intellectual property rights, the system also tends to adopt independent standards in its implementation. Therefore, their function needs to be carefully designed to provide effective support for family farm information management. The system presented in this paper uses five types of NFC tags with different functions:

(1) #A-type NFC tag: NFC tag for the in-field service node
(2) #B-type NFC tag: NFC tag for agricultural device management
(3) #C-type NFC tag: NFC tag for agriculture process management
(4) #D-type NFC tag: NFC tag for agriculture product market management
(5) #E-type NFC tag: NFC tag for agriculture value-added service

**Figure 4:**
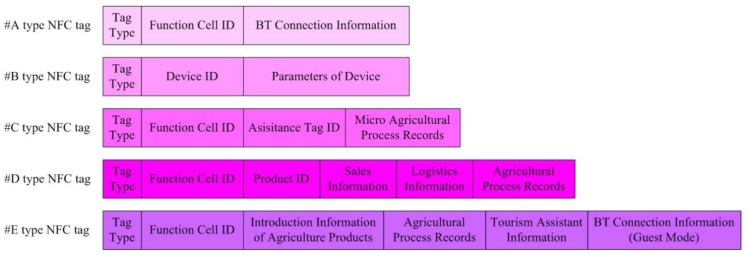
Information stored in NFC tags.

In these NFC tags, the information content is stored in encoded form. These codes have a similar format and correspond to different types of agricultural information. The conversion between codes in NFC tags and agricultural information can be done through the MA App. For cost and procurement convenience, we recommend using the NFC tags that are commonly used in the market (for example, NTAG213) for these types of functions. Especially in agricultural applications, NFC tags will encounter solar radiation, rain, and watering, among other environmental conditions. Therefore, it is recommended to use epoxy-packaged NFC tags. Due to the wide use and secure supply of ISO/IEC14443 Type A NFC labels in China, in the engineering implementation of our design, we used waterproof NTAG213 (ISO/IEC14443 Type A) with epoxy and PVC packaging. The information stored in NFC tags is shown in Figure 4.

The #A-type NFC tag is used for smartphone-enabled access to the in-field service node. In NFC tag A, the encoded Bluetooth connection information of the corresponding in-field service node in the function cell is stored. After the smartphone scans NFC tag A, the MA App implements a Bluetooth connection with the in-field service node. In each function cell, there is only one NFC tag A. Normally, the #A-type NFC tag is placed on the surface of the chassis of the in-field service node, but it can also be placed in other positions in the Bluetooth coverage range of in-field service nodes. The #A-type NFC tag is essential for the fast connection between smartphones and nodes.

The #B-type NFC tag is very important for agricultural device management. The parameters of agricultural devices (such as device ID, device type, interface type, and interface parameters) are stored in the #B-type NFC tag. Each agricultural device corresponds to one #B-type NFC tag. In the PSoC, IP cores are used to manage different agricultural equipment. The MA App reads the parameters of the agricultural devices from the #B-type NFC tag and transmits them to the in-field service node. The in-field service node triggers the corresponding IP core according to the parameters and sets the working parameters in the IP core. This enables the automatic interconnection and management of agricultural devices and nodes. After completing the connection of the device to the node, the MA App also sends the agricultural device parameters in the #B-type NFC tags to the MS App for the user application operations that are accessible through the UI.

Agricultural processes at different locations vary among the function cells. For example, because of the different distribution of solar radiation and water in the soil, the growth of tomatoes in different locations in the greenhouse will differ. As a result, there will be some differences in the fertilization, pesticide, and pruning processes (micro agricultural process records). Recording these processes can help manage the production process of the tomatoes. Therefore, the #C-type NFC tag can be placed in locations in which fine-grained agricultural process management is required. The user writes the micro agricultural management records in the agricultural process to the #C-type NFC tag by using the MS App and the MA App in the smartphone. Compared with other methods, the low cost of NFC tags makes them more suitable for intensive and refined agricultural process records. Usually, in a function cell, farmers can deploy dozens of #C-type NFC tags. It is even possible to arrange multiple #C-type NFC tags for multiple different management processes in the same location in order to record agricultural processes more granularly.

The #D-type NFC tag is usually attached to the packaging of agricultural products. Using the smartphone, the farmer obtains the agricultural product production process information stored in the #D-type NFC tag from the in-field service node and the #C-type NFC tag near the agricultural production location of a given product. Sales information and logistics information can be added to #D-type NFC tags using the MS App and MA App in the smartphone. Sales personnel and customers can scan the #D-type NFC tags with their smartphones to view the agricultural product information that is important to them. This enables transparency of the agricultural production and sales process of a product.

In today’s family farms in China, most agricultural value-added services are agricultural tourism and agricultural education. In agricultural tourism, the main profit comes from the on-site consumption of agricultural products. Agricultural education primarily involves the immersion of children and adolescents in the agricultural experience. The design of #E-type NFC tags focuses on these two services. The #E-type NFC tags store the introduction information about the agricultural products, the agricultural process records, and the Bluetooth information of the in-field service node for the guest mode. #E-type NFC tags will also contain tourism assistant information, such as positioning. The above information is written by the farmer and accessed by visitors using the revised MS app.

## 4. In-Field Service Node

In-field service nodes allocated to each function cell act as IoT access points for distributed management and service. Connecting agricultural electromechanical device and IoT equipment to the in-field service node can help the farmer to carry out distributed in-field management of these devices with the in-field service nodes as the core. For example, in the greenhouse, farmers can use Bluetooth to access nodes through NFC scanning, and then can view and control the surrounding agricultural facilities. Thus it provides a direct and effective means for farmers to directly handle in the agricultural information management and service process. And it is help to combine the actual observation and management experience of farmers to achieve a more efficient management process in daily management of agriculture in family farm. It is very suitable for the daily management habits of Chinese family farm farmers too. The management of these agricultural devices via in-field service nodes is based on the information present in the NFC tags passed by MS Apps. The agricultural devices in family farms are of various types. Some of them have standardized data interfaces, such as RS-232, RS-485, I2C, and SPI, whereas others need to be controlled via PWM or by logic signals combined with relays. Many of these devices also need analog interactions. In addition, different agricultural devices have large functional differences depending on their applications. On some occasions, the in-field service nodes must meet the needs of personnel management, macro agricultural data storage, among others. During in-field service node design, the above-mentioned diverse device management requirements should be met as much as possible and functional scalability should be provided, under a unified hardware design. The main functions of the In-field service node are:

(1) NFC-based agricultural device management
(2) Bluetooth-based smartphone access management
(3) Node power management
(4) Macro agricultural information management
(5) LoRa communication management (optional, for centralized management only)
(6) Auxiliary management function (optional)

PSoC enables a highly integrated digital–analog hybrid embedded system to realize flexible and customized functions on a silicon chip [62]. With its highly integrated CPU, storage, digital subsystem, analog subsystem, digital/analog bus, Universal Digital Block (UDB) and other system resources, it can adapt to the needs of family farm IoT information management with function differences. The biggest advantage of PSoC is that its high functional flexibility can bring application scalability to meet the diversified application needs of family farms. By combining native IP cores and UDB modules in the design, users can quickly build IP cores and development APIs for specific applications with independent intellectual property rights. Using this method, traditional businesses based on hardware platforms can be transformed into businesses based on IP core services. To meet the different requirements of agricultural devices on interfaces and functions, developers can specially combine PSoC’s internal system resources, supplemented with UDB design and IP cores of their own. This can help achieve the design with high functional flexibility and scalability on the unified hardware platform. Therefore, the needs of different family farms under a unified node hardware structure can be easily met. IP core reuse is oriented to different occasions. Developers only need to use IP core functions with the API interface depending on the IoT management service specification and do not need to conduct complicated work with regard to specific development for devices. In other words, the hardware function implementation process is simplified as a series of IP cores and APIs using. This method can also optimize the system through the addition and deletion of IP cores. In our proposed design, each IP core function structure related to a device corresponds to an NFC tag. According to the information in an NFC tag, in-field service nodes can connect with devices and imply daily management by the IP cores automatically. A PSoC has high scalability and supports the IP core-based management/service mode, the in-field service nodes designed based on PSoCs have the advantages of high adaptability, universality and reliability, short development cycle, low cost, and having independent intellectual property rights.

**Figure 5:**
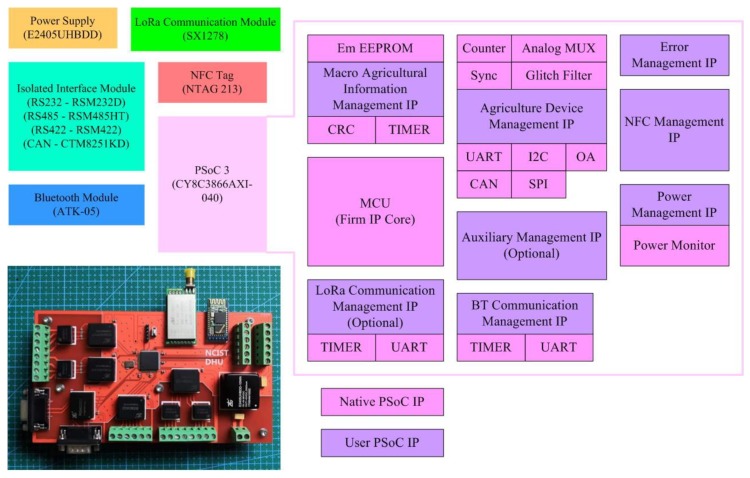
Hardware structure of in-field service node.

The in-field service node is designed based on the CY8C3866AXI-040 PSoC3 chip (Cypress Semiconductor Corp, San Jose, CA USA). The CY8C3866AXI-040 chip is built with a high-speed MCU up to 67 MHz. It also has a UDB array comprising 24 PLD-based configurable UDB units. A design combining the UDB array with the native IP cores of the PSoC chip can meet the requirements of family farms for agricultural management functions in different scenarios. For example, the node uses a BT 4.0 Bluetooth module to support smartphones. Through this Bluetooth module, NFC tag information and other agricultural management data can be exchanged between the smartphone and the node. As the Bluetooth module is connected to the node through the UART interface, the native UART of PSoC and the UDB are combined in the design to form the user IP core and API. An API can be used to manage the corresponding Bluetooth communication. After being passed into the PSoC, the NFC information is managed through an NFC device management IP core built based on the UDB, thus realizing corresponding NFC related functions in the in-field service node. Based on IP cores for NFC device management, agricultural devices with standard digital interfaces can be related to IP cores that can be dynamically configured on the CY8C3866AXI-040 chip. To realize interfaces under non-standard protocols, logic values or PWM, and analog signals, user IP cores can be built using native IP cores and the corresponding modules of the PSoC chip (such as UDB, GPIO PWM, and operational amplifier with dynamic programmable configurations). The communication process mode between an in-field service node and agricultural devices can be set to the query mode or the autonomous transmission mode based on internal timing. In the query mode, the in-field service node sends query instructions to a device based on the serial number depending on the scheduled time round or other management logic. After receiving the instruction, the device executes the data operations and control operations. In the autonomous transmission mode, a device sends data to the node according to predetermined control logic, which is set by the user in the NFC tag of the device. The above two functions are realized through the corresponding user IP core function structures. The hardware structure of in-field service node is shown in Figure 5.

The energy consumption management IP core of the node can optimize energy consumption based on the sleep–wake mechanism and accordingly monitors the power supply of the node. Macro agricultural information is encoded and stored in the internal storage space of PSoC and managed by the IP core for macro agricultural information management. In the design of CY8C3866AXI-040 IP core for family farm optional management service, there are auxiliary management IP core and LoRa communication management IP core, which support people management and LoRa communication, among others. When the PSoC of the in-field service node exchanges information with external devices, protocol conversion (such as UART-RS232) is required in some cases. In addition, electromagnetic shock may be introduced due to data cables or the surrounding environment. Therefore, we used electromagnetic isolation interfaces and surge suppressors in the design to solve these problems. We also used the DC–DC power module with high conversion efficiency and electromagnetic isolation for the in-field service node.

## 5. Management Assistant App and Management Service App

### 5.1. App Configuration in Smartphone

Combining smartphones with IoT access nodes will not only realize management functions such as agricultural in-field monitoring and control but also support in-field service functions such as agricultural tourism. However, due to various types of agriculture devices, extensive agricultural application requirements, strong difference of application functions, and other related factors, the development of a full-function application covering hardware support to customized service for information management in each family farm faces great challenges with regard to R&D cost, development cycle, development complexity, among other aspects.

The MA App can bridge the underlying hardware with practical applications and solve the above problems to a large extent. In MA Apps, we encapsulate the native Android functions related to the underlying hardware and common agricultural business. The MA App also provides easy-to-use interfaces to application-oriented MS Apps. The developers of application-oriented MS Apps can focus on function development without having to pay more attention to the underlying hardware operation and Android function operation. Application layer-oriented developers can realize MS Apps with multiple functions based on the unified MA App. The MA App exchanges information with in-field service nodes through normalized data containers. This not only improves the reliability of information interaction between smartphones and nodes but also increases convenience and scalability for hardware and application developers. If the hardware or other support structure is changed, only the MA App needs to be modified without modifying the MS App. This facilitates the maintenance of the system and the need for continuous informationization construction for Chinese family farms. In addition, separating the MA App from the MS App facilitates different information service companies to perform the corresponding business mode. For example, some small companies and individual developers can focus on the development of MS App, while some large-scale companies can focus on MS App and the corresponding underlying hardware development. In the system, the function mode between MA Apps and in-field service nodes is the trigger-response mode. The function mode between the MS App and the MA App is the master–slave mode with request and feedback. The difference between the above-mentioned two modes is that the management functions of MS Apps are basically built on MA Apps, while the operations between MS Apps and in-field service nodes rely more on the collaboration between hardware and software.

### 5.2. Management Assistant App

The MA App plays a bridging role for node hardware and application. It encapsulates the operation of hardware devices and Android agricultural management service-related functions and can be used for applications through simple interface. In the cooperation between an MA App and an in-field service node, it is usually the MA App that sends operation information to the in-field service node and receives feedback from the node. The MA App does not intervene in the specific execution process of the in-field service node. The main functions of the management assistant App are:

(1) NFC tag management
(2) NFC-based Bluetooth communication management
(3) Location information management
(4) Hierarchical interface management
(5) User Management
(6) Data management
(7) Error management

NFC tag management is the main function of the MA App. NFC tag management of the MA APP does not include only NFC tag reading and writing based on system application requirements. The corresponding relationship between the encoded information stored in the NFC tag and the actual description is also stored in a table in the MA App. After the MA App obtains the encoded information in the NFC tag, it converts the information into the corresponding binary management description. NFC-based Bluetooth communication management in MA Apps can create and manage Bluetooth connections between in-field service nodes and smartphones with the assistance of NFC tags. #A-type NFC tags and #B-type NFC tags are mainly oriented to hardware operations related to in-field service nodes. The latter three types of NFC tags are mainly aimed at the requirements generated by the MS Apps. Therefore, the former uses the real-time mode in management, while the latter uses the request mode in processing. In the real-time mode, when the NFC tag is recognized, the corresponding process of MA App is immediately triggered. In the request mode, only after an MS App issues a tag reading request to an MA App, the MA App will conduct the corresponding operation.

Location information management of MA Apps provides location information in terms of longitude and latitude for MS Apps. The hierarchical interface management of MA Apps is used to manage the interfaces between MA Apps, in-field service nodes, and MS Apps. The interface data among MA Apps is in the Socket format, whereas the interface data between MA Apps and in-field service nodes is in the Bluetooth packet format. User management provides MS Apps with user login, authentication, security management, and other services. Data management implements data storage, data connection, and other services required for agriculture information management. MA Apps can also manage NFC tag errors, Bluetooth communication errors, interface errors, and Android function errors that may occur.

### 5.3. Management Service App

There are differences between the management and service demands of family farms in China. In this system, the function scalabilities of in-field service nodes and MA Apps can adapt to the above situation. In this manner, the MS App can use the above structure to meet the diversified agricultural information management service needs of family farms. Depending on the different requirements of family farm management, MS Apps can be built on a combination of the following main functions. The main functions of the management service App may be:

(1) User Interface
(2) Graphical display of data
(3) NFC tag application management
(4) Device application management
(5) Data application management
(6) Map service
(7) WeChat service

**Figure 6:**
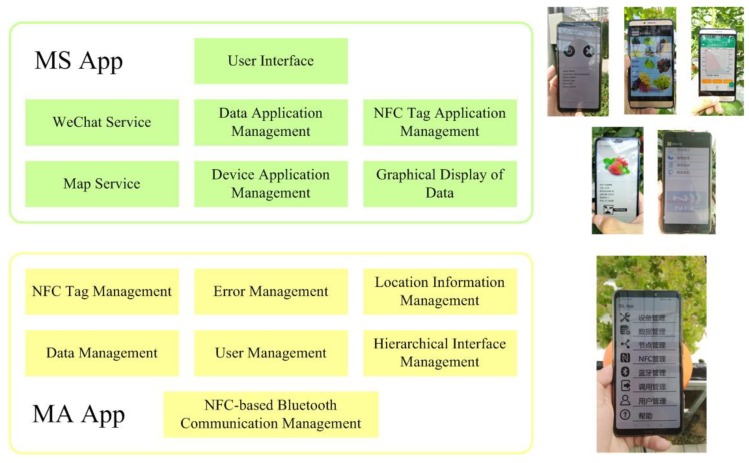
MA App and MS App.

A user interface should be adopted to provide intuitive and effective human–smartphone interaction to users. Graphical display of data can visually display family farm agricultural management service data. NFC tag application management can provide NFC-related services to users (for example, NFC-based agricultural product traceability services). Users can manage the agricultural devices that have been connected to the in-field service nodes through MS Apps. They can also realize management and service based on the agricultural data. All the above functions are based on MA Apps and in-field service nodes. In addition, MS Apps can provide map service and push service for users based on third-party map apps (Baidu Maps or Google Maps) and WeChat. It should be noted that users can flexibly choose and combine the above functions according to their needs. The ultimate goal of a good MS App is to meet the actual agricultural management needs. Developers of the MS App should avoid adding unrelated functions to the MS Apps if there are no corresponding management services in the family farm. The functions of MA App and MS App are shown in Figure 6.

## 6. System Application in Family Farms

After the completion of the system, it was put into use on five family farms in Hebei Province and Jiangsu Province. These family farms, which were selected as pilots for the system applications, have typical agricultural operations, are self-employed, and have typical agricultural management characteristics. In addition, the agricultural management data of these farms is comprehensive, which provided us with favorable conditions for evaluating the effectiveness of the system. The characteristics and business of these farms are shown in Table 1.

These family farms are mainly engaged in high-yield vegetables and fruit trees. The operators have an urgent need for information management in their agricultural production processes. In particular, through the introduction of the management service system, it is necessary to fine-tune the agricultural production process, increase the output of agricultural products, reduce labor costs, and further increase operating income. The agricultural devices on these family farms are mainly oriented to irrigation, sensing, and greenhouse operations. In the greenhouse-related management service especially, the number of devices involved is higher, and the degree of refinement of the agricultural process management is also higher. What is beneficial to the system is that most of the equipment has standardized interfaces, driven by the standardization of agricultural information equipment in China in recent years. Due to the government’s policy orientation, most family farms carry out agricultural value-added services based on agricultural tourism. In addition, not all family farm operators want to use remote central management. Many family farm operators prefer to use their smartphones to visually manage agricultural processes in the field or greenhouse. They are especially interested in using intuitive NFC tags as the basis for management. This feature is also reflected in the system design. When using the proposed system, these family farms are first divided into a series of function cells based on agricultural functions and management needs. Each function cell corresponds to a greenhouse or a parcel that needs to be managed. Usually, there are more function cells for a family farm operating mainly in greenhouses.

The application of the proposed system in the family farms is shown in Table 2.

In order to meet the actual management needs of these family farms, we designed an MS App based on the unified in-field service node hardware and the MA APP. In the test, the system node and the smartphone APP ran stably and reliably. Pre-defined IP cores in the in-field service node effectively supported device connections based on NFC tags. With NFC tag operation, farmers can quickly establish a connection between the node and their smartphone. During the test, there were no Bluetooth communication errors that could not be handled by the MA APP. Devices can be quickly added to nodes via the MS APP and NFC tags. After addition, the IP core-based device management service can be effectively executed. Since the planting variety and planting process of the family farm may change frequently, agricultural devices need to be reconfigured easily. The NFC-based device connection function has great practicality. Family farms have some differences in the use of NFC tags for agricultural planting process management. Some farms focus on recording the planting process in agricultural areas, while others use planting varieties and planting service types for tag installment. The architecture of the system can effectively meet these needs. Family farms can also flexibly use NFC tags to provide information services for agricultural products, based on the needs of the dealer. According to feedback from the dealers, the satisfaction of the seller and the customer with their agricultural products increased. Different family farms offer different ways of providing value-added services. The use of NFC tags in some family farms for value-added services is product-oriented. The labels are often placed near specialized products or their services.

The value-added services of other family farms are more macroscopic. It may be more desirable to allow visitors to experience agricultural farming processes, rather than focusing on agricultural products. The NFC tags are used more densely in the former case. The latter situation requires a more elaborate design of the service flow for the NFC tag. Through the system, these two requirements were also effectively met.

In addition, under the requirements of centralized management in some family farms, nodes can effectively send data to the corresponding interface of the host computer through the LoRa low-power wide-area network. Therefore, centralized management services can be implemented. Macro-agricultural parameter storage (such as temperature in a greenhouse) and personnel attendance can also be effectively supported under the functional support of in-field service nodes. The system effectiveness in in the family farms is shown in Table 3.

In order to evaluate the effectiveness of the system, we conducted evaluations from the perspective of agricultural process management, agricultural product business, and agricultural value-added services. We mainly evaluated agricultural process management by measuring the yield and input of agricultural products in the same length of time. The vegetables and fruits in the evaluation were measured by the weight of the yield, while the poultry products were measured by the quantity. Relatively speaking, the full production cycle of greenhouse agricultural products is relatively short. The production cycle of field vegetables and poultry products is much longer. In the evaluation, the evaluation period covered at least one production cycle of the agricultural product. In the survey of tourists, sellers and customers, a questionnaire based on a five-point scale was used. It can be seen from the evaluation results that after adopting the system for agricultural management, the yield of greenhouse agricultural products was more obvious, and the output of outdoor cultivated vegetables, fruit trees and poultry products was also improved to some extent. The system effectively optimized agricultural inputs. For greenhouse agriculture and field planting, the water, pesticides, fertilizers, and labor inputs consumed by the agricultural process were reduced to some extent. The most important reason for the above is the refinement of the rough agricultural management method on the family farm based on the NFC label management process. The farm operators can refine the details of the agricultural management, and optimize it. Cost input in various agricultural processes can be refined in this way too. The evaluation results of agricultural product satisfaction show that sellers and customers reflect a greater increase in satisfaction with the agricultural products, despite there not being much difference in the quality and variety of agricultural products produced before and after the system was put into operation. The main reason for this increase in satisfaction was that the transparency of agricultural production processes enabled customers to understand factors of concern for the safety of agricultural products, thus promoting consumption. In addition, some sellers also provided targeted services based on agricultural process information, logistics information and prices in the NFC tags, which also increased the satisfaction of buyers and sellers. The evaluation of agricultural value-added services was based on income evaluation and a questionnaire of user satisfaction of agricultural value-added services. Four of the five family farms had agricultural value-added services before the system was used. Compared with historical data, it can be seen that after the application of the system, the agricultural value-added service income and the agricultural value-added service satisfaction were significantly improved. The most important reason for this factor is that visiting tourists could effectively obtain agricultural information from the NFC tag. The various process details in the agricultural process led to a significant increase in the recognition of agricultural products. Visitor also received better service through the NFC tags on the family farms. This increased their consumer desire and consumer satisfaction. All in all, the application of the system on various family farms shows that it effectively meets the IoT information construction needs of family farms. It effectively improves the operational efficiency of family farms, and provides support for their long-term development.

## 7. Discussion

The proposed design mainly realizes the construction of an agricultural IoT information management service system for family farms that utilize NFC. Using the process of separating the support structure from the application, as previously described, the application-oriented application (App) does not require the creation of a functional support structure for the underlying hardware and the support function of a smartphone in the development. Thus, the development cycle is significantly shorter.

Studies have shown that using Internet of Things (IoT) technology as the main supporting technology for an agricultural IoT informationization upgrade can effectively improve agricultural production efficiency, hence increasing farmer income. However, for family farms in China, due to the limited scope of IoT technology in regard to practitioners, and the limited capital investment and limited resources, targeted design is needed to meet their special requirements. In recent years, the promotion of smartphones in China has laid a solid foundation for the popularization of agricultural information technology, based on the IoT. Near-field communication (NFC) technology provides an appropriate means of fundamentally meeting the practical needs of agricultural management and services, based on the IoT technology utilized in the information construction of Chinese family farm. As a storage medium, NFC tags are easy to embed in various agricultural scenes and are easy to enter into the market alongside agricultural products. This enables full process management services in agricultural production. The NFC tag arrangement is intuitively embedded in agricultural applications, whereby the user can intuitively establish a connection between the NFC tag and the associated object through visual means and experience. Farmers and tourists can use their smartphones directly to participate in agricultural process management and agricultural services. Its low cost also contributes to its large-scale adoption in family farms in China.

Providing agricultural information management functions, based on NFC, for family farms utilizing IoT is an efficient scheme. However, if NFC based design is applied to specific management practices of Chinese family farms, two other problems primarily need to be solved. The first problem is the requirement for an IoT information terminal hardware structure that can work effectively with NFC tags. Second, there is a need for information services, implemented on a smartphone that can meet the diverse needs of Chinese family farms based on NFC tags. Both of the hardware and APP need to have extremely strong functional scalability. Moreover, costs and ease of use also require more careful consideration. This poses a major challenge to the system design. In the system proposed in this paper, PSoC and MA App structure are used to separate the hardware structure and the smartphone function support structure from the application support structure, for use on a family farm. Using this proposed solution, the need to provide information services to diverse applications using the underlying hardware and software support under a unified structure is realized. After the system was completed, experimental use was carried out using family farms that exhibited typical characteristics. These family farms include greenhouse cultivation, field planting, and poultry farming. In use, the systems functional scalability was fully reflected. Based on hardware, MA App and MS App, it fully met the needs of the family farms. The agricultural production efficiency of family farms, agricultural product sales and value-added services all increased considerably.

Since NFC tags can also be used as sensors [63,64]. In future work, NFC-based sensors can be considered for the system to further enhance the system’s performance. The use of these new sensing technologies can enhance the monitoring of agricultural processes, and it is also easy to achieve real-time tracking of agricultural product quality. The family farm management process can be further refined and optimized. And it also helps to generate new business models. Developers can rely on the powerful computing and information interaction capabilities of smartphones to provide deeper services based on the proposed system design. Additionally, adding decision support and machine learning functions in the MS App is suggested, thus providing a higher level for agricultural operators. In the MS APP, the corresponding functions are added to help the agricultural process management to combine the wisdom of agriculture experts and the personal experience of family farm operators, and to achieve more optimized family farm information management. For agricultural management departments, large-scale agricultural information monitoring and management services can also be built by adding interfaces for cloud information services in the MS App. On the hardware side, the PSoC’s IP core structure can also be used to provide adaptation to agricultural equipment manufacturers, thus enabling direct plug-in and direct use on the hardware platform. In the business model reliant on the system, not only can the agricultural production operators and service providers bring profits through an IoT informationization upgrade of the original agricultural equipment and the corresponding value-added services, but also the IP core and the core development of the corresponding equipment of such an enterprise. The MA App adapts to generate revenue and form a sustainable profit model. Therefore, a similar design of the system can be expected to have greater market potential; hence, the corresponding business model is worth exploring and implementing.

## 8. Patents

Three Chinese patents (Chinese Patent Number: 2017211523064, 2016111355942, and 2014101686206) and one software copyright (Chinese Software Copyright Registration Number: 2018SR938365) resulted from the work reported in this manuscript.

## Figures and Tables

**Table 1:** The characteristics and business in the family farms.

	Family Farm #1	Family Farm #2	Family Farm #3	Family Farm #4	Family Farm #5
Agricultural Method	Greenhouse	Field	Greenhouse and Field	Field and Coop	Greenhouse
Main Agricultural Products	Vegetables	Vegetables and Fruit	Vegetables and Fruit	Vegetables and Poultry	Fruit
Agriculture Device	Irrigation Device (Pump, Valve), Sensor (T/H, Soil, Solar Radiation), Greenhouse Device (Fan, Motor, Light)	Irrigation Device (Pump, Valve), Sensor (T/H, Soil, Wind)	Irrigation Device (Pump, Valve), Sensor (T/H, Soil, Wind), Greenhouse Device (Fan, Motor)	Irrigation Device (Pump, Valve), Sensor (T/H, Soil)	Irrigation Device (Pump, Valve), Sensor (T/H, Soil, Solar Radiation), Greenhouse Device (Fan, Motor, Door)
Agriculture Device Interface	RS-232,RS-485, PWM, Analog	1-wire, RS-232,RS-485, PWM	1-wire, RS-232,RS-485, PWM, Analog	Logic Level, RS-232,RS-422, RS-485, PWM	1-wire, RS-232,RS-485, PWM, Analog
Value-add Service	Agriculture Tourism	Agriculture Tourism	Agriculture Tourism and Agriculture Education	None ^1^	Agriculture Tourism
Need for Human Resource Management	No	No	Yes	No	Yes
Need for Central Management	No	Yes	Yes	Yes	No

^1^ This family farm does not provide agricultural tourism services due to business and license reasons.

**Table 2:** The application of the proposed system in the family farms.

	Family Farm #1	Family Farm #2	Family Farm #3	Family Farm #4	Family Farm #5
Number of Function Cells	17	13	21	14	19
Number of In-field Service Node	17	8 ^1^	14 ^1^	12	11 ^1^
#A-type NFC tag	13	10	14	12	11
#B-type NFC tag	37	24	51	29	42
#C-type NFC tag	135	217	294	194	122
#D-type NFC tag ^2^	800+	300+	700+	500+	400+
#E-type NFC tag	40	10	32	None	34
Main Scope of MS App	Greenhouse Management and Tourism Service	Planting Management and Tourism Service	Greenhouse Management, Planting Management and Tourism/Education Service	Agricultural Process Management (Vegetables and Poultry)	Greenhouse Management and Tourism Service

^1^ These family farms did not use the system in all function cells for the purpose of staging construction. ^2^ Only available at requests.

**Table 3:** System effectiveness in in the family farms ^1^.

	Family Farm #1	Family Farm #2	Family Farm #3	Family Farm #4	Family Farm #5
Yield ^1,2^	[10%, 15%]	[0%, 5%]	[15%, 20%] in Greenhouse [5%, 10%] in Field	[10%, +15%] in Greenhouse No obvious increase in Poultry	[5%, 10%]
Water ^1,3^	[5%, 10%]	[5%, 10%]	[15%, 20%] in Greenhouse [10%, 15%] in Field	[10%, +15%] in Greenhouse	[10%, 15%]
Pesticides and Fertilizers ^1,4^	[20%, 25%]	[0%, 5%]	[10%, 15%] in Greenhouse [0%, 5%] in Field	[10%, +15%] in Greenhouse	[10%, 15%]
Labor ^1,5^	[20%, 25%]	[10%, 15%]	[15%, 20%] in Greenhouse [5%, 10%] in Field	[15%, 20%] (Overall Statistics)	[25%, 30%]
Agricultural Product Satisfaction (Customs)	Increased from 3.8 to 4.7	Increased from 4.0 to 4.3	Increased from 3.7 to 4.5	Increased from 4.3 to 4.5	Increased from 3.5 to 4.5
Agricultural Product Satisfaction (Sellers)	Increased from 4.4 to 4.6	No Obvious Increase	Increased from 3.4 to 4.7	Increased from 4.4 to 4.5	Increased from 4.3 to 4.6
Value-add Service Satisfaction	Increased from 4.2 to 4.9	Increased from 4.1 to 4.4	Increased from 4.0 to 4.6	No Value-add Service	Increased from 4.1 to 4.4
Increase of Value-add Service Income	34%	15%	27%	No Value-add Service	17%

^1^ The statistics of agricultural data are often subject to certain deviations due to the influence of environmental and details of agricultural behavior. So in the table, we use 5% intervals to represent results that cannot be accurately counted. ^2^ The yield statistics of agricultural products are easily affected by drying, transportation, man-made loss, etc. So, the yield statistics of vegetables and fruits are based on the weight after harvesting. ^3^ Based on water supply records. ^4^ Based on use records. ^5^ Statistics based on attendance data.
